# In vitro and in vivo biocompatibility of a porcine cholecystic extracellular matrix (CECM) membrane for tissue regeneration

**DOI:** 10.1038/s41405-025-00370-4

**Published:** 2025-10-09

**Authors:** Betcy Thomas, Thomas George Velliavettil, Kanakarajan V. Pratheesh, Mekha Grace Varghese, Rani Shine Raju, Yogesh Bharat Dalvi, Sukumaran Anil, Nibu Varghese, Avneesh Chopra, Nebu George Thomas

**Affiliations:** 1 Department of Periodontology, Pushpagiri College of Dental Sciences, Thiruvalla, Kerala India; 2 Experimental Pathology, Biomedical Technology Wing, Sree Chitra Tirunal Institute for Medical Sciences and Technology, Thiruvananthapuram, Kerala India; 3 Pushpagiri Research Centre, Pushpagiri Institute of Medical Science and Research Centre, Pushpagiri Medical Society, Thiruvalla, Kerala India; 4 Department of Dentistry, Oral Health Institute, Hamad Medical Corporation, Doha, Qatar; 5 Department of Conservative Dentistry and Periodontology, Medizinische Hochschule Brandenburg (MHB) Theodor Fontane, Brandenburg an der Havel, Germany; 6 Department of Periodontology, Oral Medicine and Oral Surgery, Institute for Dental and Craniofacial Sciences, Charité–University Medicine Berlin, Corporate member of Freie Universität Berlin, Humboldt–Universität zu Berlin, and Berlin Institute of Health, Berlin, Germany

**Keywords:** Dental biomaterials, Periodontitis

## Abstract

**Background:**

Periodontal disease affects 3.5 billion people globally, resulting in annual treatment costs exceeding $54 billion. Guided tissue regeneration (GTR) membranes are essential for periodontal therapy, but commercially available options often suffer from limitations, including high cost, limited accessibility in resource-limited settings, and suboptimal mechanical properties. This study aimed to develop and characterize a novel porcine cholecystic extracellular matrix (CECM)-based GTR membrane and comprehensively evaluate its physicochemical properties, cytocompatibility, and in vivo biocompatibility compared to the commercially available Healiguide® membrane.

**Methods:**

CECM membranes were fabricated through systematic decellularization, lyophilization, and ethylene oxide (ETO) sterilization of porcine gallbladders. Surface characterization was performed using scanning electron microscopy (SEM) with quantitative pore analysis, and biochemical composition was assessed via Fourier Transform Infrared Spectroscopy (FTIR). MTT assays were performed on L929 fibroblast cells to evaluate cytocompatibility. Wound healing capacity was assessed using scratch assays monitored over 72 h. In vivo biocompatibility was evaluated through subcutaneous implantation in Sprague-Dawley rats, with histological analysis performed at 1, 2, 3, and 4 weeks post-implantation.

**Results:**

SEM analysis revealed that CECM membranes exhibited a heterogeneous, multilayered structure with larger average pore sizes compared to Healiguide® (18.2 ± 4.6 µm vs. 12.5 ± 3.2 µm, *p* < 0.05), facilitating enhanced cellular infiltration. FTIR confirmed the preserved integrity of collagen in both membranes, with CECM showing an enhanced glycoprotein content indicative of retained bioactive components. Cytocompatibility assessment demonstrated excellent cell viability for CECM, showing 97.4 ± 1.6%, 94.2 ± 1.8%, and 90.8 ± 1.4% viability at 20, 50, and 100 µg/mL CECM extracts, respectively. The scratch assay demonstrated superior wound healing capacity for CECM, with significantly enhanced wound closure at 72 h compared to Healiguide® (89.7 ± 6.1% vs. 79.4 ± 5.8%, *p* < 0.05). Subcutaneous implantation studies confirmed excellent in vivo biocompatibility, with CECM showing lower initial inflammatory response (inflammation score: 2.3 ± 0.5 vs 2.8 ± 0.6 at week 1, *p* < 0.05), enhanced vascularization (12.3 ± 2.1 vs 9.7 ± 1.8 vessels/hpf at week 3, *p* < 0.05), and superior tissue integration compared to commercial controls.

**Conclusion:**

The porcine CECM membrane demonstrated favorable physicochemical properties, excellent cytocompatibility, enhanced wound healing potential, and superior tissue integration characteristics compared to commercial GTR membranes. These preliminary findings provide a strong scientific foundation supporting the development of the GTR membrane for periodontal regenerative therapy.

## Introduction

The periodontium, consisting of the gingiva, periodontal ligament, cementum, and alveolar bone, forms the functional unit that supports the teeth. Periodontitis is a chronic inflammatory disease that progressively destroys these supporting structures, leading to periodontal attachment loss and bone resorption [1]. This destruction can ultimately lead to tooth loss, resulting in masticatory dysfunction, compromised esthetics, and negative impacts on self-confidence and overall quality of life. Periodontal disease represents a significant global health challenge, affecting approximately 3.5 billion people worldwide according to the Global Burden of Disease Study 2019 [2]. Globally, it is the 11th most prevalent disease, affecting 11.2% with its severe form [3, 4]. The economic burden of periodontitis is substantial, with global treatment costs exceeding $54 billion annually [5]. In developing countries like India, epidemiological data reveal a prevalence of periodontal disease at 51%, highlighting the critical need for accessible and cost-effective treatment modalities [6].

Achieving periodontal health involves eliminating infection, resolving inflammation, and preventing disease recurrence. While traditional periodontal surgeries improve clinical outcomes, they primarily facilitate tissue repair rather than proper regeneration of cementum, periodontal ligament, and alveolar bone. Reconstructive surgical techniques, such as guided tissue regeneration (GTR), use barrier membranes, bone grafts, and biological mediators to promote periodontal regeneration. An ideal GTR membrane must be biocompatible, non-toxic, flexible, and biodegradable to eliminate the need for surgical removal while providing adequate mechanical support during the healing period. Natural membranes, such as collagen-based matrices derived from biological sources, offer excellent biocompatibility and support for tissue regeneration [7]. These membranes act as natural scaffolds, primarily biocompatible occlusive barriers that maintain space while promoting new tissue formation and vascularization. By preventing the infiltration of unwanted cells, such as epithelial cells, into the healing site, they allow the desired cells, like periodontal ligament cells and osteoblasts, to regenerate the damaged tissues.

Collagen-based GTR membranes have gained attention due to their natural resorbability, biocompatibility, and hemostatic properties, which facilitate wound healing [8]. Type I collagen membranes, which are natural and resorbable, can be obtained from the tendon, dermis, skin, or pericardium of human, porcine, or bovine origin. In addition to their excellent biocompatibility, these membranes possess beneficial characteristics such as low immunogenicity and the ability to increase tissue thickness [9]. However, challenges persist, including reduced mechanical strength, rapid resorption, and potential disease transmission [10], particularly with bovine sources due to concerns about prion diseases such as bovine spongiform encephalopathy (BSE) [11]. These limitations and inconsistent degradation rates restrict the clinical applicability of collagen-based membranes.

While porcine-derived materials may raise concerns regarding immunogenicity and cultural acceptance in some populations, porcine collagen remains the most clinically validated and widely available xenogenic source for GTR applications [12]. In some studies, alternative sources of type I collagen, such as fish skin or eggshell membranes, have demonstrated lower immunogenic and allergic responses, but further clinical validation is required [9]. Cultural and religious considerations may limit acceptance of porcine materials in specific populations, though they remain widely accepted in most clinical settings.

To enhance the mechanical stability and mitigate the rapid resorption of collagen matrices, various physical, chemical, and enzymatic cross-linking methods have been employed, including ultraviolet radiation, glutaraldehyde, diphenylphosphoryl azide, and hexamethylene diisocyanate. These methods aim to prolong the degradation time and enhance the mechanical properties of collagen membranes, addressing current material deficiencies [13]. However, cross-linked collagen matrices face limited clinical use due to biocompatibility, tissue integration, and biodegradation concerns, particularly with glutaraldehyde-induced and enzymatic cross-linking. Excessive cross-linking can also affect water absorption and membrane stiffness, impacting clinical usability [14, 15].

Another type of natural membrane used in periodontal regeneration is the acellular dermal matrix (ADM) membrane, such as Alloderm®. These membranes maintain the extracellular matrix (ECM) structure, including collagen, elastin, and endogenous growth factors, supporting host tissue integration. After decellularization, ADM membranes act as scaffolds for host tissue ingrowth, promoting tissue regeneration [16, 17]. However, the high cost and regulatory restrictions set by the Drug Controller General of India (DCGI) limit their availability in regions like India, creating a significant gap in accessible treatment options for periodontal regeneration.

An innovative alternative to conventional ADM membranes is decellularized porcine cholecystic extracellular matrix (CECM) membranes, which have demonstrated significant regenerative potential in animal models, particularly for skeletal muscle, subcutaneous tissue, and skin wound repair [18]. CECM contains key components, including type I collagen, sulfated glycosaminoglycans, elastin, and growth factors such as fibroblast growth factor (FGF), vascular endothelial growth factor (VEGF), and transforming growth factor-beta (TGF-β), all of which are crucial for tissue regeneration [19]. Previous research has demonstrated that CECM membranes offer excellent osteoconductivity, biocompatibility, and support for cell adhesion, proliferation, and differentiation, making them a promising and cost-effective alternative for tissue regeneration in periodontal and other regenerative applications [20]. The decellularization process effectively removes cellular antigens while preserving beneficial ECM components, which potentially reduces immunogenicity concerns associated with xenogenic materials.

Using porcine CECM represents an innovative approach in periodontal regeneration, with significant potential as a biomaterial in regenerative medicine. Given the global burden of periodontal disease, the limitations of current treatment options, and the need for cost-effective solutions, particularly in resource-limited settings, there is a critical need for developing accessible GTR membrane alternatives. This study aimed to develop and characterize a porcine CECM-based GTR membrane and evaluate its physicochemical properties, cytocompatibility, and in vivo biocompatibility as a preliminary step toward potential clinical translation for periodontal regeneration applications. The study compared the novel CECM membrane with Healiguide®, a commercially available porcine collagen membrane widely used in clinical practice [21, 22], to provide a relevant benchmark for performance evaluation. The goal was to establish the scientific foundation for a cost-effective, locally manufactured GTR membrane that could improve access to periodontal regenerative therapy, particularly in developing countries where commercial membrane costs may be prohibitive.

## Materials and methods

### Fabrication process of the porcine CECM-based GTR membrane

The fabrication process of the porcine CECM-based GTR membrane is shown in Appendix Fig. 1. Fresh porcine gallbladders were obtained from Meat Production of India (MPI), Kottayam, Kerala, India, and incubated in 10% neutral buffered formalin for 24 h. After rinsing with tap water, the bile was released by cutting open both ends (fundus and apex), and the bile content was removed under running tap water. The mucosal layer of the gallbladder was carefully peeled off to avoid damage, followed by decellularization and the removal of the muscular layer by scraping [23]. The tissue was then placed in tissue cassettes and stored in a plastic container filled with distilled water at −80 °C for 16 h. The fibro-muscular layer cassette was washed under moderate-flow tap water for 4–6 h. After washing, four membrane layers were spread on a glass plate and vacuum-pressed using another glass plate. The membranes were then lyophilized overnight (16 h) in a lyophilizer. The dried membranes were packaged in sterilization rolls and laminated using a photo-laminator to ensure uniform thickness. The sheets were then cut into rectangular shapes (10 mm × 10 mm × 0.5 mm), sealed in sterilization rolls, and subjected to ethylene oxide (ETO) sterilization. This method was selected to ensure effective sterilization while preserving the structural integrity of the collagen matrix, as confirmed by subsequent SEM and FTIR characterization experiments that demonstrated maintained morphological and biochemical properties post-sterilization.

#### Control membrane

Healiguide® (Advanced Biotech Products (P) Ltd., Tamil Nadu, India) was selected as the control membrane due to its widespread clinical use as a commercially available porcine collagen-based GTR membrane [21, 22], providing a relevant benchmark for comparison with our novel CECM membrane. This choice was further justified by the need to compare against a clinically validated standard with established safety and efficacy profiles, while maintaining consistency in source material (porcine origin) to eliminate species-related variables and focus on processing-related differences between the membranes. The absence of a separate negative control group was justified, as our study design focused on a comparative evaluation between the experimental CECM membrane and the clinically established Healiguide® standard, following conventional biomaterial evaluation protocols. This approach allows for comparison to approved commercial materials, which provides more clinically relevant data than non-implanted controls.

### Physicochemical characterization

#### Morphological analysis by scanning electron microscopy (SEM)

The decellularized porcine CECM membrane and Healiguide® control were washed in chilled phosphate-buffered saline (PBS) (0.1 mol/L, pH 7.2) and then fixed in 2.5% glutaraldehyde in PBS for 6 h. The fixed membranes were dehydrated in a graded series of acetone solutions (30%, 50%, 70%, 80%, 90%, 95%, and 100%) at 4 °C for 15 min each, followed by three washes with PBS. The samples were sputter-coated with fine gold using a DII-29030SCTR Smart Coater (fully automated). The cross-sectional morphology was examined using a JSM-7610F Plus ultra-high-resolution field emission SEM (JEOL, USA) at 15 kV with a working distance of 6.0 mm. Pore size analysis was conducted using ImageJ software (NIH, Bethesda, MD), measuring at least 100 randomly selected pores per sample (*n* = 3 independent samples per group). Structural integrity was assessed by evaluating collagen fiber organization, multilayered architecture, and membrane continuity across multiple magnification levels.

#### Chemical composition analysis by Fourier transform infrared-attenuated total reflectance (FTIR-ATR)

The chemical composition of both porcine CECM and Healiguide® was analyzed using FTIR-ATR. Approximately 2 mg of lyophilized samples were mixed with dry potassium bromide in a 1:10 ratio and pressed into flakes. FTIR spectra were obtained using a Shimadzu 8400S spectrophotometer, scanning from 4000 to 400 cm⁻¹ with 32 scans per spectrum. The analysis was performed at room temperature (25°C) in a dry atmosphere. Three independent samples were analyzed for each group (*n* = 3). The resulting spectra were analyzed using OMNIC software (Thermo Fisher Scientific) to identify and characterize absorption peaks associated with collagen structures.

### Cell culture assays

For in vitro cell assays, the L929 mouse fibroblast cell line was obtained from the National Centre for Cell Sciences (NCCS), Pune, India, and cultured in Dulbecco’s Modified Eagle’s Medium (DMEM) supplemented with 10% fetal bovine serum (FBS), L-glutamine, sodium bicarbonate, and an antibiotic solution containing penicillin (100 U/mL), streptomycin (100 µg/mL), and amphotericin B (2.5 µg/mL). Cells were incubated at 37 °C in a humidified 5% CO_2_ incubator overnight (18–24 h) before further experiments (NBS Eppendorf, Germany).

#### Cytotoxicity analysis (MTT assay)

The cytotoxicity of the membranes was evaluated according to ISO 10993-5 guidelines [24]. L929 cells were seeded at a density of 1 × 10⁴ cells per well in 96-well plates and incubated for 24 h at 37 °C with 5% CO_2_ to achieve ~90% confluence before testing. Following ISO 10993-5 guidelines, the extract-based approach was chosen to evaluate potential leachable cytotoxic substances, representing the standard method for initial biocompatibility screening of medical devices. While this conservative approach ensured regulatory compliance and standardized safety assessment, we acknowledge that future direct contact studies would provide complementary information about cell-membrane surface interactions and mechanotransduction effects.

After reaching confluence, the medium in each well was replaced with extracts containing substances released from the test (CECM) groups at three different concentrations (20 µg/mL, 50 µg/mL, and 100 µg/mL), prepared in complete DMEM medium. The test extracts were obtained by incubating membrane samples in complete medium for 24 h at 37 °C, followed by filtration and dilution to the desired concentrations. These concentrations were selected following ISO 10993-5 guidelines for dose-dependent cytotoxicity evaluation. Complete DMEM medium was used as the negative control (100% viability). Each concentration was tested in triplicate wells across three independent experiments (*n* = 9 total measurements per concentration per group), and the plates were incubated for 24 h. The MTT assay measures mitochondrial cellular metabolism (viability) and estimates the number of viable cells. After the experiment, the culture was washed with 1× PBS, and 15 µL of MTT solution per mL of culture medium (MTT 5 mg/mL dissolved in PBS and filtered through a 0.2 µm filter) was added. The plates were incubated at 37 °C for 3 h, and 200 µL of Dimethyl sulfoxide (DMSO) was added to each well. The contents were then incubated at room temperature for 30 min until all cells were lysed and a homogeneous color was obtained. The solution was centrifuged for 2 min to sediment cell debris. The optical density (OD) was measured spectrophotometrically at 540 nm using a microplate reader (BioTek, Winooski, VT). Cells treated with the MTT solution without the sample served as the control. Cell viability was calculated as follows: Cell viability (%) = (OD of treated cells/OD of control cells) × 100.

#### Scratch assay

Exponentially growing L929 cells were trypsinized and seeded at a density of 2 × 10⁵ cells per well in 12-well plates for 24 h to reach ~90% confluence. The medium was removed, and a scratch was made along a pre-marked line using a sterile 1 mL pipette tip. After removing debris from the scratches, the monolayer was rinsed three times with PBS and incubated with CECM and Healiguide® extracts, prepared at 20 µg/mL, for 24, 48, and 72 h. Each treatment was performed in triplicate across three independent experiments (*n* = 9 total per time point per group). Images were taken at the intersection of the scratched areas and the pre-marked lines using an inverted microscope (×100 magnification, Olympus CKX41) at 0-, 24-, 48-, and 72-h post-scratch. The effect on wound closure was measured using ImageJ analysis software (NIH, Bethesda, MD), and wound contraction was calculated using the following formula:

$${{{\rm{Wound}}}}\; {{{\rm{contraction}}}}\,( \% )= 	 [({{{\rm{Initial}}}}\; {{{\rm{gap}}}}\; {{{\rm{width}}}}-{{{\rm{Final}}}}\; {{{\rm{gap}}}}\; {{{\rm{width}}}})/ \\ 	 {{{\rm{Initial}}}}\; {{{\rm{gap}}}}\; {{{\rm{width}}}}]\times 100$$

The initial gap width represents the wound width at 0 h, and the final gap width represents the wound width at 24, 48, and 72 h.

### In vivo biocompatibility

#### Animal procurement and hosting

Twenty-four male Sprague-Dawley rats, aged 8–10 weeks and weighing 150–200 g, were selected for in vivo experiments. The animals were obtained from the Kerala Veterinary College, Mannuthy, India. Sample size was determined using G*Power software (version 3.1.9.7) with α = 0.05, power = 0.8, and effect size = 1.2, yielding *n* = 6 per group per time point. This calculation resulted in 24 male Sprague-Dawley rats (*n* = 12 per treatment group, with *n* = 6 animals sacrificed at each of the four time points: 1-, 2-, 3-, and 4 weeks post-implantation). The effect size of 1.2 was chosen to detect clinically meaningful differences in inflammatory response, tissue integration, and vascularization parameters between the CECM and Healiguide® groups. This sample size provided sufficient statistical power while adhering to the 3Rs principle (Replacement, Reduction, Refinement) of animal research ethics by using the minimum number of animals necessary to achieve statistically valid results.

Housing and in vivo experiments were conducted under standard laboratory conditions at the Laboratory Animal Care (LAC) Facility, Pushpagiri Institute of Medical Sciences and Research Centre, Thiruvalla, Kerala, India. The rats were maintained under a 12-h artificial light/dark cycle, at a constant room temperature of 22 °C, and a relative humidity of approximately 52%. They were fed a conventional laboratory diet of rat/mouse pellets supplied by Krish Scientist’s Shoppe, Bangalore, India. All procedures, including animal sacrifice, anesthesia, surgery, and postoperative care, were performed in a conventional aseptic environment to ensure animal welfare and minimize potential risks or complications.

Ethical approval for the animal experiments was obtained from the Institutional Animal Ethics Committee (IAEC), Pushpagiri Institute of Medical Sciences and Research Centre, Thiruvalla, Kerala, India (Approval Number: PIMS/IAEC/2023/04). The study was conducted strictly following the guidelines and regulations of the Committee for Control and Supervision of Experiments on Animals (CPCSEA), constituted by the Animal Welfare Division of the Government of India. Animal experiments were conducted and reported according to the ARRIVE (Animal Research: Reporting of In Vivo Experiments) guidelines.

#### Subcutaneous scaffold implantation and tissue extraction

The animals were randomly divided into two groups, with 12 rats assigned to the porcine-derived CECM group and 12 to the commercially available Healiguide® group. Anesthesia was induced by intraperitoneal injection of ketamine hydrochloride (40–100 mg/kg) and xylazine (5–13 mg/kg). The dorsal surface of each rat was aseptically prepared using 10% (w/v) povidone-iodine solution, followed by epilation. A subcutaneous injection of 1 mL of 0.9% sodium chloride solution was administered to ensure hydration. Standardized rectangular scaffolds measuring 10 mm × 10 mm × 0.5 mm were implanted through 8 mm dorsal incisions created after aseptic preparation and local hydration. The sterilized scaffolds were carefully implanted into the subcutaneous pocket, ensuring proper positioning and contact with surrounding tissues. Incisions were closed with non-absorbable Ethicon surgical sutures, ensuring appropriate tension without compromising tissue perfusion.

Postoperatively, the animals were closely monitored by the animal care staff for three days. During this period, ceftriaxone (25–50 mg/kg) and meloxicam (0.5 mg/kg) were administered intramuscularly for pain management and infection prevention. Daily monitoring included an assessment of the surgical wound, food consumption, activity levels, and clinical signs of infection.

Rats were humanely euthanized by CO_2_ inhalation at 1-, 2-, 3-, and 4-week post-implantation (*n* = 6 per group per time point). The scaffolds and surrounding tissues were carefully removed and immediately immersed in PBS. Tissue sections containing the scaffolds were photographed, sectioned, and fixed in 10% formalin for 48 h. After fixation, the samples were preserved in 70% ethanol, embedded in paraffin, and sectioned for histological analysis. Sections were stained with hematoxylin and eosin (H&E) and examined under a light microscope (×40 magnification), with observations documented photographically [25, 26]. Histological evaluation was performed by a qualified pathologist, blinded to the treatment groups, using a semiquantitative scoring system adapted from previously validated criteria [27] by ISO 10993-6 standards for assessing local tissue responses to implanted materials [28].

### Statistical analysis

Statistical in vitro and in vivo studies were analyzed using GraphPad Prism software (GraphPad Software, Inc., version 9.0). All in vitro experiments were conducted with *n* = 3 independent biological replicates. For SEM analysis, *n* = 3 independent samples per group were analyzed with quantitative assessment of at least 100 pores per sample. FTIR analysis was performed on *n* = 3 independent samples per group. MTT assays were conducted as *n* = 3 independent experiments, each performed in triplicate (*n* = 9 total per concentration per group). Scratch assays were performed as *n* = 3 independent experiments in triplicate (*n* = 9 total per time point per group). Data are presented as mean ± standard deviation (SD). A *p*-value < 0.05 was considered statistically significant. The Shapiro-Wilk test was used to assess the normality of the data, which indicated that most data did not follow a normal distribution. Therefore, the Kruskal-Wallis test (non-parametric equivalent of one-way ANOVA) was used to compare differences between groups, followed by Dunn’s post-hoc test for multiple comparisons. For the scratch assay time-course data, two-way ANOVA was used because this dataset met the assumptions for parametric testing (normality and equal variances) and involved repeated measures over time, followed by Tukey’s multiple comparison test for post-hoc analysis.

## Results

### SEM analysis reveals comparable porous architecture between CECM and Healiguide® membranes

**Fig. 1:**
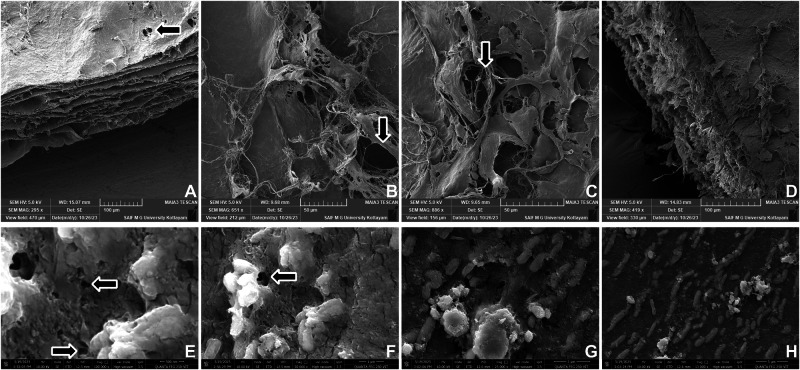
Comparative ultrastructural analysis of CECM and Healiguide® membranes. SEM images of the decellularized porcine CECM membrane (**A**–**D**) and the commercial Healiguide® control (**E**–**H**) at varying magnifications. **CECM**: **A** Low-magnification overview showing multilayered lamellar architecture (295×, scale bar = 100 µm). **B** Intermediate magnification displaying an interconnected collagen fiber network and pores (651×, scale bar = 50 µm). **C** Higher magnification highlighting pore microstructure (886×, scale bar = 50 µm). **D** Cross-sectional view with fiber density and surface integrity at low magnification (419×, scale bar = 100 µm). Healiguide®: **E** High magnification view (100,000×, scale bar = 500 nm) revealing compact collagen arrangement and surface porosity. **F** Medium magnification (50,000×, scale bar = 1 µm), showing heterogeneous fiber clusters. **G**, **H** Energy dispersive X-ray (EDX) elemental mapping of surface mineral deposition (25,000× and 13,000×, scale bars = 3 µm and 5 µm, respectively). Representative pores are indicated with arrows (→) showing typical size ranges for each membrane type. Images demonstrate the multilayered structure, porous network, and surface topography essential for cell attachment and nutrient diffusion. *n* = 3 independent samples per group.

SEM analysis of the decellularized porcine CECM membrane and Healiguide® control (Fig. 1) revealed distinct morphological characteristics. The CECM membrane (Fig. 1A–D) displayed a sheet-like surface with slight wrinkling and a distinct multilayered structure. At lower magnifications, an interconnected network of collagen fibers formed a matrix with evenly distributed pores, predominantly ranging from 10 to 25 µm in diameter (average: 18.2 ± 4.6 µm, *n* = 100 pores analyzed across 3 independent samples). High magnification images further highlighted the layered arrangement of collagen and the presence of micro-pores (2–5 µm), underscoring the ideal surface topology for cell attachment and proliferation. The Healiguide® control membrane (Fig. 1E–H) showed a more uniform fibrous structure with smaller average pore sizes (12.5 ± 3.2 µm, *n* = 100 pores analyzed across 3 independent samples) and a more compact arrangement of collagen fibers. Both membranes demonstrated structural integrity and maintained their collagenous nature after processing. However, the CECM membrane showed greater porosity and a more heterogeneous architecture that may facilitate enhanced cell infiltration and nutrient exchange. Representative pores are indicated with arrows (→) showing typical size ranges for each membrane type, with CECM displaying larger interconnected pores compared to the more compact Healiguide® structure.

### FTIR analysis confirms preserved collagen structure in both CECM and Healiguide® membranes

**Fig. 2:**
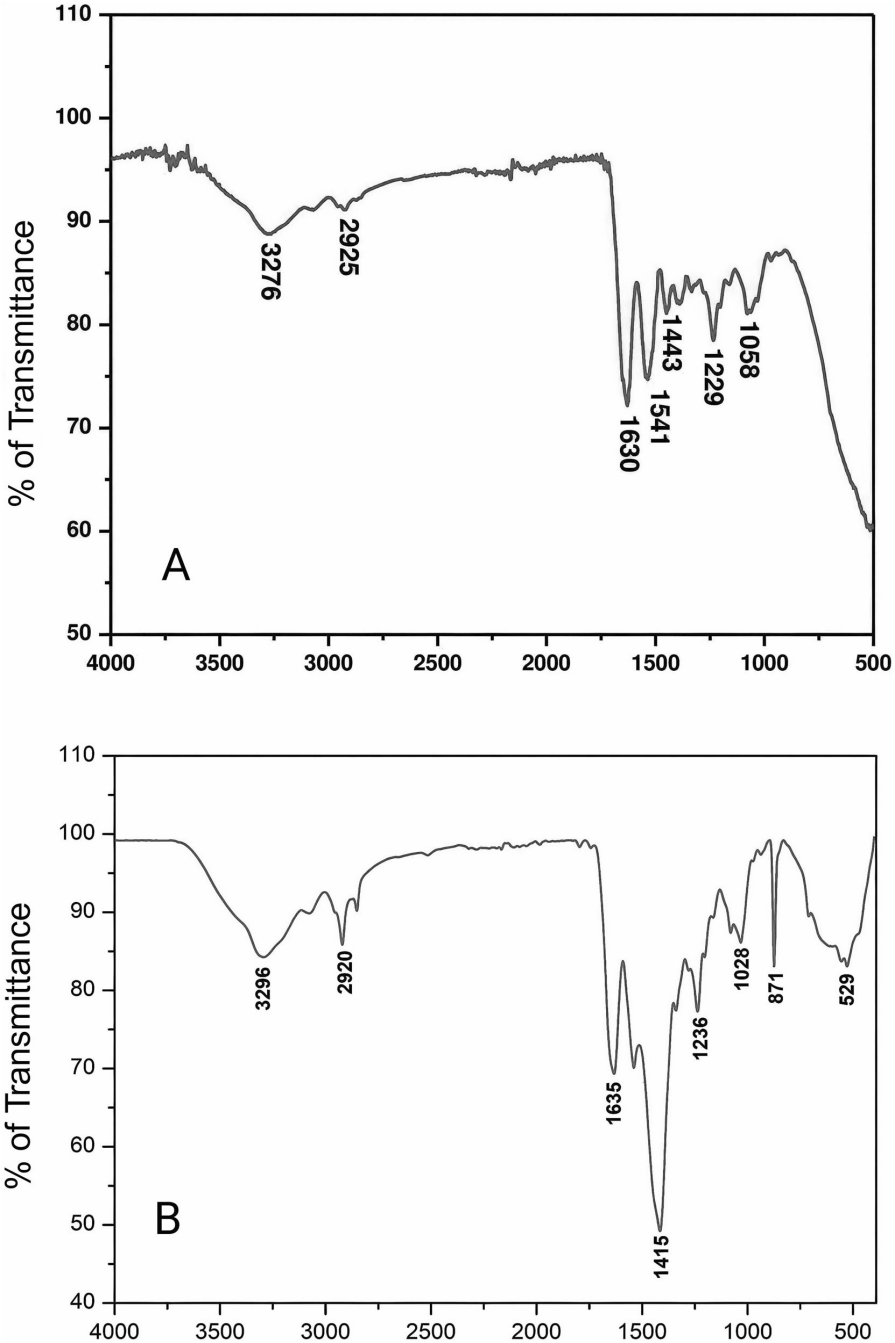
FTIR analysis demonstrating biochemical composition of porcine CECM and Healiguide® membranes. FTIR spectra of the decellularized porcine CECM membrane (**A**) and the commercial Healiguide® control (**B**) are presented. Characteristic amide peaks (A, B, I, II, and III) and glycoprotein groups are labeled, confirming preservation of collagen integrity and presence of bioactive components in both membranes. The inset shows a magnified view of the glycoprotein region (1000–1300 cm⁻¹), highlighting group differences. Representative spectra from *n* = 3 independent samples per group are shown. No statistical analysis was performed on spectral data, as this represented qualitative biochemical characterization to confirm structural preservation.

Comparative FTIR analysis of the porcine CECM membrane and Healiguide® (Fig. 2A, B) revealed similar protein functional groups crucial for biological activity, confirming successful preservation of collagen structure in both materials. Representative FTIR spectra from *n* = 3 independent samples per group are shown. Qualitative comparison revealed similar collagen-associated peaks in both groups, with CECM showing enhanced glycoprotein content as evidenced by peaks at 1229 and 1058 cm⁻¹. No statistical analysis was performed on spectral data, as this represented qualitative biochemical characterization to confirm structural preservation.

Three major spectral regions were observed in both groups:

Region 1 (3200–3500 cm⁻¹): The amide A band, related to N–H stretching vibration typical of intermolecular hydrogen bonding, was observed at 3276 cm⁻¹ for porcine CECM and 3285 cm⁻¹ for Healiguide®. The amide B band, associated with symmetric stretching of CH₂, was found at 2925 cm⁻¹ for CECM and 2931 cm⁻¹ for Healiguide®.

Region 2 (1500–1700 cm⁻¹): The amide I peak, characteristic of C=O stretching vibrations in proteins, appeared at 1630 cm⁻¹ for CECM and 1635 cm⁻¹ for Healiguide®. The amide II band, related to C–N stretching and N–H in-plane bending from amide linkages, was present at 1541 cm⁻¹ for CECM and 1545 cm⁻¹ for Healiguide®.

Region 3 (1000–1500 cm⁻¹): The amide III band was located at 1443 cm⁻¹ for CECM and 1448 cm⁻¹ for Healiguide®, confirming hydrogen bonding preservation. Notable differences were observed in the glycoprotein region. CECM showed distinct peaks at 1229 cm⁻¹ and 1058 cm⁻¹ attributed to C–O–C stretching vibrations in collagen-associated glycoproteins, which were less pronounced in Healiguide®. These enhanced glycoprotein peaks in CECM indicate retention of bioactive extracellular matrix components that support cell adhesion, proliferation, and tissue integration, contributing to the superior regenerative potential observed in our biological assays.

These results confirm that both membranes maintained their structural integrity and biochemical potential for regenerative applications, with CECM showing enhanced glycoprotein content that may contribute to its biological activity.

### MTT assay demonstrates excellent cytocompatibility of CECM scaffolds

**Fig. 3:**
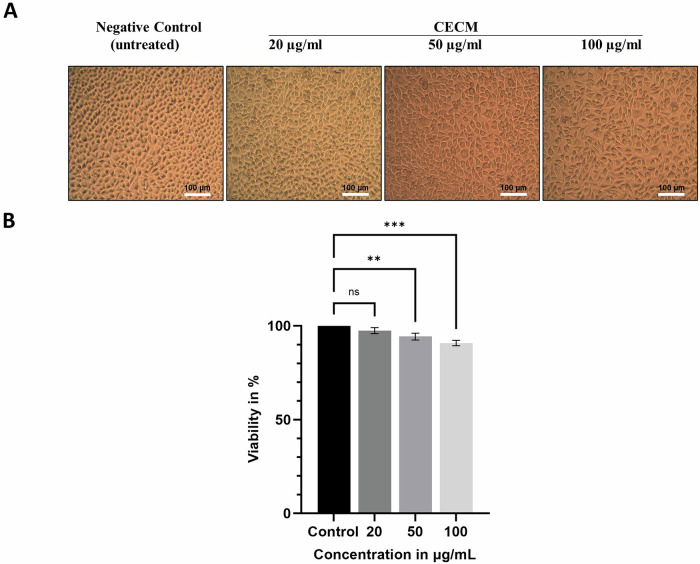
Cytocompatibility assessment of porcine CECM membranes. **A** Representative phase-contrast images of L929 fibroblast cells showing normal cell morphology after 24-h exposure to CECM membrane extracts at different concentrations. Two groups are shown: negative control (untreated) and CECM Extract. Scale bars: 100 µm. **B** Quantitative MTT assay results showing percentage cell viability at 20, 50, and 100 µg/mL concentrations with standard deviation error bars. Data presented as mean ± SD from *n* = 3 independent experiments performed in triplicate (*n* = 9 total per concentration per group). Statistical analysis: Kruskal-Wallis test followed by Dunn’s post-hoc test. ***p < 0.001, **p < 0.01, *p < 0.05 compared to control; ns = not significant.

The cytotoxicity evaluation of porcine CECM scaffolds at 20, 50, and 100 µg/mL concentrations using the MTT assay on L929 fibroblasts over 24 h demonstrated excellent biocompatibility (Fig. 3). Phase-contrast microscopy revealed normal cell morphology with no signs of cytotoxicity in both the negative control (untreated) and CECM extract-treated groups (Fig. 3A). Cell viability remained high across all tested concentrations: 97.4 ± 1.6%, 94.2 ± 1.8%, and 90.8 ± 1.4% at 20, 50, and 100 µg/mL respectively, compared to the untreated control (100% viability) (*n* = 3 independent experiments, each performed in triplicate, total *n* = 9 per concentration per group) (Fig. 3B). These findings indicate that both membranes provide favorable environments for cell growth, with cell viabilities consistently above 90% according to ISO 10993-5 cytotoxicity standards.

### Enhanced wound healing capacity of CECM membrane in scratch assay

**Fig. 4:**
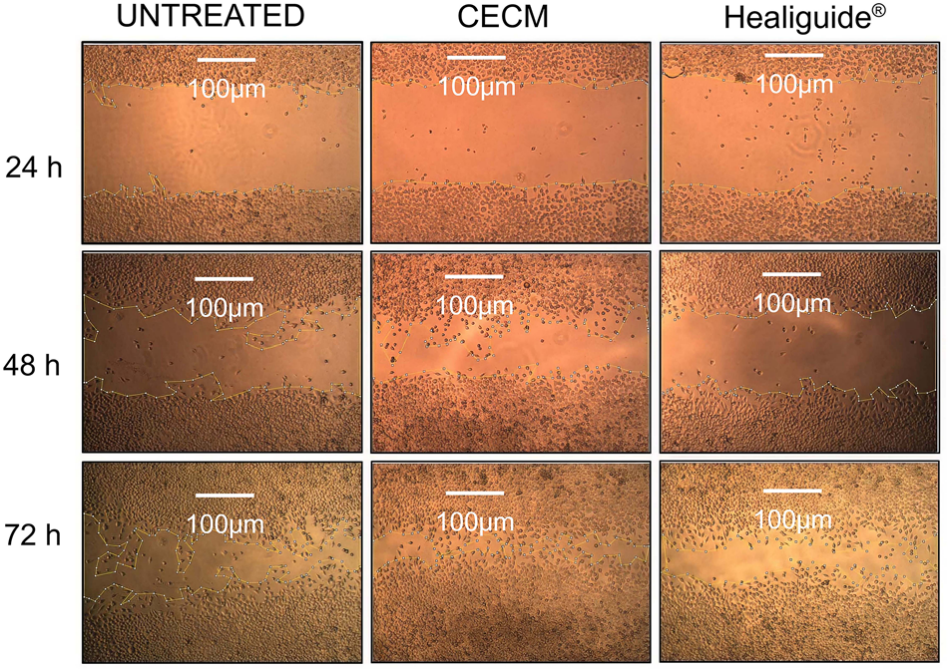
Wound healing assessment using scratch assay. Representative images showing wound closure progression at 24, 48, and 72 h for three groups: negative control (untreated), CECM membrane, and Healiguide® (commercial comparator). Scale bars: 100 µm. Quantitative analysis shows percentage wound closure over time, demonstrating the superior healing capacity of the CECM membrane. Data presented as mean ± SD from *n* = 3 independent experiments performed in triplicate (*n* = 9 total per time point per group). Statistical analysis: Two-way ANOVA followed by Tukey’s multiple comparison. ***p < 0.001, **p < 0.01, *p < 0.05.

The scratch assay results (Fig. 4) demonstrated superior wound healing properties of the decellularized porcine CECM membrane compared to both Healiguide® and the untreated control. The three groups evaluated were: untreated control (negative control), Healiguide® extract, and CECM extract. Wound closure rates were significantly enhanced in the CECM group across all time points:

- 24 h: CECM: 32.5 ± 4.2%, Healiguide®: 28.1 ± 3.8%, Control: 24.6 ± 3.1%
- 48 h: CECM: 68.9 ± 5.7%, Healiguide®: 58.3 ± 4.9%, Control: 51.2 ± 4.5%
- 72 h: CECM: 89.7 ± 6.1%, Healiguide®: 79.4 ± 5.8%, Control: 72.8 ± 5.2%

Statistical analysis revealed significant differences between CECM and control groups at all time points (*p* < 0.01, two-way ANOVA) and between CECM and Healiguide® at 48 and 72 h (*p* < 0.05). This enhanced healing response is likely attributed to bioactive growth factors and enhanced collagen architecture retained in the CECM matrix, facilitating faster fibroblast migration and proliferation.

### Excellent in vivo biocompatibility and progressive tissue integration of CECM membrane

**Fig. 5:**
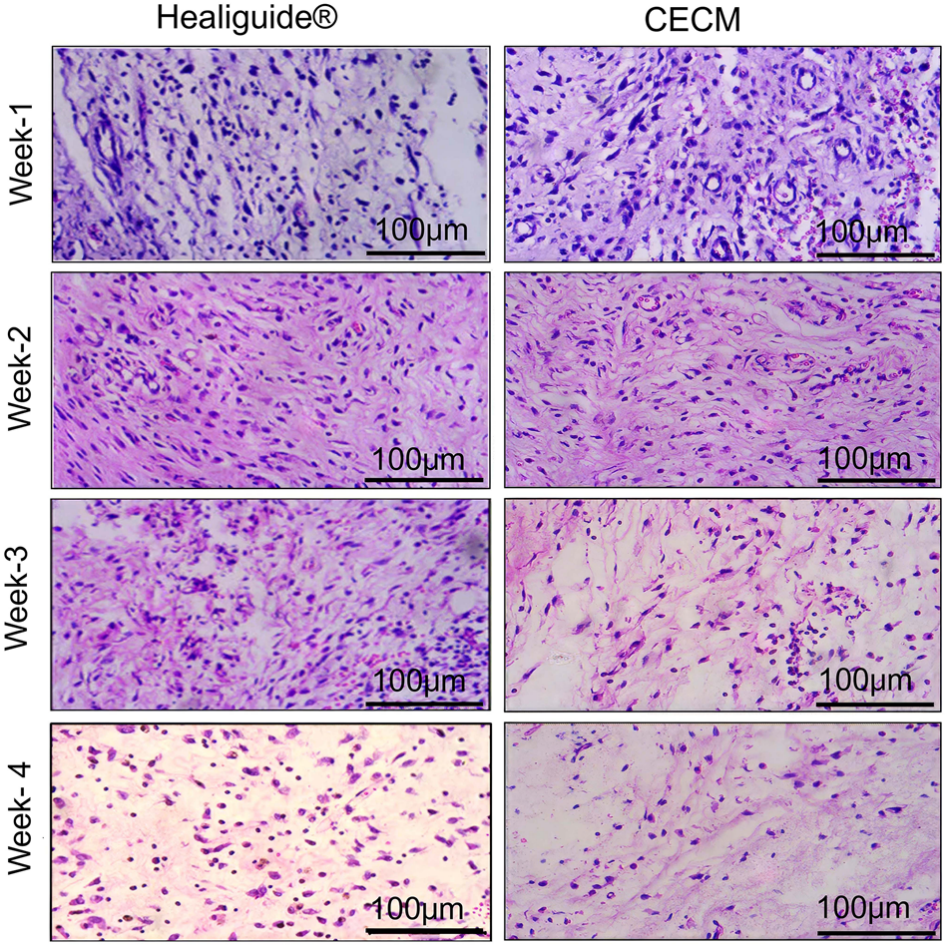
Histological analysis of tissue response to implanted membranes. Representative H&E-stained sections comparing Healiguide® (commercial control) and CECM groups at 1, 2, 3, and 4 weeks post-implantation. Low-magnification overview images (×40 magnification with 100 µm scale bars) depict the membrane–tissue interface at subcutaneous implantation sites. Quantitative analysis was performed by a blinded pathologist (*n* = 6 animals per group per time point).

Histological analysis of implanted scaffolds over four weeks demonstrated excellent biocompatibility and progressive tissue integration of the CECM membrane (Fig. 5). Quantitative assessment by a blinded pathologist revealed distinct temporal patterns in both Healiguide® and CECM groups:

Week 1: Both groups showed typical acute inflammatory responses with moderate polymorphonuclear neutrophil and eosinophil infiltration. The CECM group exhibited slightly lower inflammatory cell counts compared to Healiguide® (inflammation score: CECM 2.3 ± 0.5 vs Healiguide® 2.8 ± 0.6, *p* < 0.05).

Week 2: Progressive reduction in acute inflammatory cells was observed in both groups, with increased presence of macrophages and lymphocytes. CECM sites showed earlier onset of fibroblast infiltration compared to control (fibroblast density: CECM 145 ± 23 cells/hpf vs Healiguide® 112 ± 18 cells/hpf, *p* < 0.05).

Week 3: Both groups showed normalized tissue architecture with minimal inflammatory response. CECM sites demonstrated enhanced vascularization with increased capillary density (CECM: 12.3 ± 2.1 vessels/hpf vs Healiguide®: 9.7 ± 1.8 vessels/hpf, *p* < 0.05).

Week 4: Complete tissue integration was observed in both groups, with CECM showing superior collagen deposition and tissue organization. The CECM group demonstrated significantly higher numbers of mature fibroblasts and enhanced matrix remodeling compared to Healiguide® (mature collagen score: CECM 3.8 ± 0.4 vs Healiguide® 3.2 ± 0.5, *p* < 0.05).

Throughout the study period, no animals showed signs of implant rejection, systemic toxicity, or adverse reactions, confirming the excellent biocompatibility of both membranes. The progressive transition from acute inflammation to tissue regeneration and integration demonstrates the CECM membrane’s superior capacity for supporting tissue healing and regeneration.

## Discussion

The primary therapeutic goals in periodontal disease management include eliminating microbial infection and promoting periodontal regeneration, achievable through various methods such as root surface conditioning agents, bone grafts, and GTR-based applications [29]. However, indigenous technologies are limited for developing low-cost GTR membranes, particularly in developing countries. With periodontal disease affecting 3.5 billion people globally and global treatment costs exceeding $54 billion annually [2, 5], the need for accessible alternatives is paramount. Our results suggest that porcine CECM membranes could provide a viable solution to this global health challenge [30, 31]. We performed the first comprehensive evaluation of a novel porcine CECM membrane as a potential GTR biomaterial, demonstrating favorable physicochemical properties, excellent cytocompatibility, and superior wound healing characteristics compared to the commercially available Healiguide® membrane. Our findings provide crucial preliminary evidence supporting the development of cost-effective, locally manufactured GTR membranes that could address the significant global burden of periodontal disease, particularly in resource-limited settings where current treatment options remain economically inaccessible.

The SEM analysis revealed distinct architectural differences between the CECM and Healiguide® membranes that have important implications for clinical performance. The CECM membrane demonstrated a more heterogeneous, multilayered structure with larger average pore sizes (18.2 ± 4.6 µm) compared to Healiguide® (12.5 ± 3.2 µm), which may facilitate enhanced cell infiltration, nutrient exchange, and tissue integration. This porous architecture is crucial for successful GTR applications, as it must strike a balance between mechanical barrier function and permeability to facilitate cellular migration and nutrient diffusion [32]. Previous research has established that optimal pore sizes for tissue engineering scaffolds typically range from 10 to 25 µm for fibroblast infiltration [33]. Our CECM membrane falls within this optimal range, suggesting superior potential for supporting periodontal regeneration compared to more compact architectures.

FTIR analysis confirmed that both CECM and Healiguide® preserved key collagen-associated functional groups essential for biological activity [34]. The presence of amide A, B, I, II, and III bands in both membranes indicated intact collagen structure, supporting their suitability for regenerative use [35, 36]. CECM showed stronger peaks at 1229 and 1058 cm⁻¹, corresponding to collagen-associated glycoproteins, which were less pronounced in Healiguide®. These glycoproteins play a key role in cell-matrix interactions and may contribute to the enhanced biological performance of CECM observed in later assays. While no statistical analysis was performed, the qualitative differences suggest that the higher retention of glycoproteins in CECM could improve regenerative potential. Overall, both membranes maintained biochemical integrity, with CECM showing possible advantages in ECM preservation.

The MTT assay confirmed excellent cytocompatibility of the porcine CECM scaffold, with fibroblast viability consistently above 90% at all tested concentrations. L929 fibroblast cells were employed for this assay, as they are the standard cell line recommended by ISO 10993-5 standards for evaluating the cytotoxicity of medical devices and biomaterials. In the in vitro scratch assay, the CECM extract significantly outperformed both Healiguide® and the untreated control in promoting wound closure across all time points. The accelerated healing observed in the CECM group is likely due to the retention of bioactive ECM components, including glycoproteins and growth factors [37] that enhance fibroblast migration and proliferation. The significant differences at 48 and 72 h indicate a sustained biological effect, underlining the therapeutic advantage of CECM in soft tissue repair contexts. These findings suggest that the decellularized porcine CECM membrane exhibits superior biochemical properties, a larger surface area, higher surface roughness, and enhanced cell adhesion and proliferation, making it an excellent candidate for GTR applications.

Histological examination over four weeks confirmed that both membranes were biocompatible; however, the porcine CECM membrane demonstrated superior regenerative performance. In the first week, an acute immune response was observed in all groups, with increased polymorphonuclear neutrophils and few eosinophils, consistent with normal post-implantation inflammation. Notably, the CECM group exhibited less pronounced inflammatory reactions compared to the commercial Healiguide®. By the second week, granulocyte counts decreased in the CECM group, with a mild presence of macrophages and lymphocytes, and reduced neutrophils, indicating an early resolution of acute inflammation. By the third week, tissue architecture began to normalize, with minimal inflammatory cells and abundant fibroblasts observed in loose connective tissue. By week four, the CECM group showed reduced macrophage numbers, increased fibroblast proliferation, and well-organized collagen deposition, indicating advanced matrix remodeling. No signs of implant rejection, systemic toxicity, or adverse reactions such as edema, discharge, or distress were observed in any animals throughout the study. This progressive transition from inflammation to healing highlights the in vivo safety and regenerative potential of the CECM membrane. Earlier fibroblast infiltration, enhanced vascularization, and superior tissue organization further underscore its bioactivity. These results strongly support the CECM membrane as a biocompatible and bioactive alternative to conventional membranes for guided tissue regeneration.

While porcine-derived materials may raise concerns about immunogenicity and cultural acceptance, our in vivo studies demonstrated minimal inflammatory response compared to commercial controls. This is because the decellularization process effectively removes cellular antigens while preserving beneficial ECM components. Future research could explore alternative sources, such as fish-derived collagen or synthetic materials; however, porcine collagen remains the most clinically validated xenogenic source for GTR applications.

### Study limitations

Several limitations of this study should be acknowledged. First, this represents a preliminary biocompatibility assessment that establishes the foundation for clinical translation but does not directly demonstrate periodontal regenerative efficacy. Future studies should include periodontal defect models to evaluate regenerative outcomes specific to periodontal applications. Second, while our in vivo assessment provided valuable biocompatibility data, longer-term studies (12–24 weeks) would better assess long-term integration and degradation characteristics. While following ISO standards, extract-based cytotoxicity testing should be supplemented with direct contact studies to better understand cell-membrane interactions. Mechanical property testing also provides essential information about membrane handling characteristics and space-maintaining capacity during clinical use.

## Conclusion

This comprehensive evaluation demonstrates that porcine CECM membranes represent a promising alternative to commercial GTR membranes, offering favorable physicochemical properties, excellent cytocompatibility, enhanced wound healing capacity, and superior tissue integration characteristics. The comparable or superior performance to commercial standards and potential cost advantages position CECM as a viable solution for addressing the global periodontal disease burden, particularly in resource-limited settings. This study’s structural characteristics, biochemical composition, and biological performance provide a strong scientific foundation for advancing CECM membranes toward clinical application. While further research is needed to optimize processing parameters and demonstrate periodontal-specific efficacy, our findings support the continued development of this indigenous biotechnology as a cost-effective solution for periodontal regenerative therapy.

## Supplementary information

Appendix Figure 1

Appendix Figure 2

Appendix Figures legends

## Data Availability

This published article and its supplementary information file contain data generated and analyzed to support this study.
