# Supplementary figures and images for: In vitro and in vivo biocompatibility of a porcine cholecystic extracellular matrix (CECM) membrane for tissue regeneration
Source: BDJ Open. 2025 Oct 9;11:81. doi: 10.1038/s41405-025-00370-4 (PMC12511280; doi:10.1038/s41405-025-00370-4)

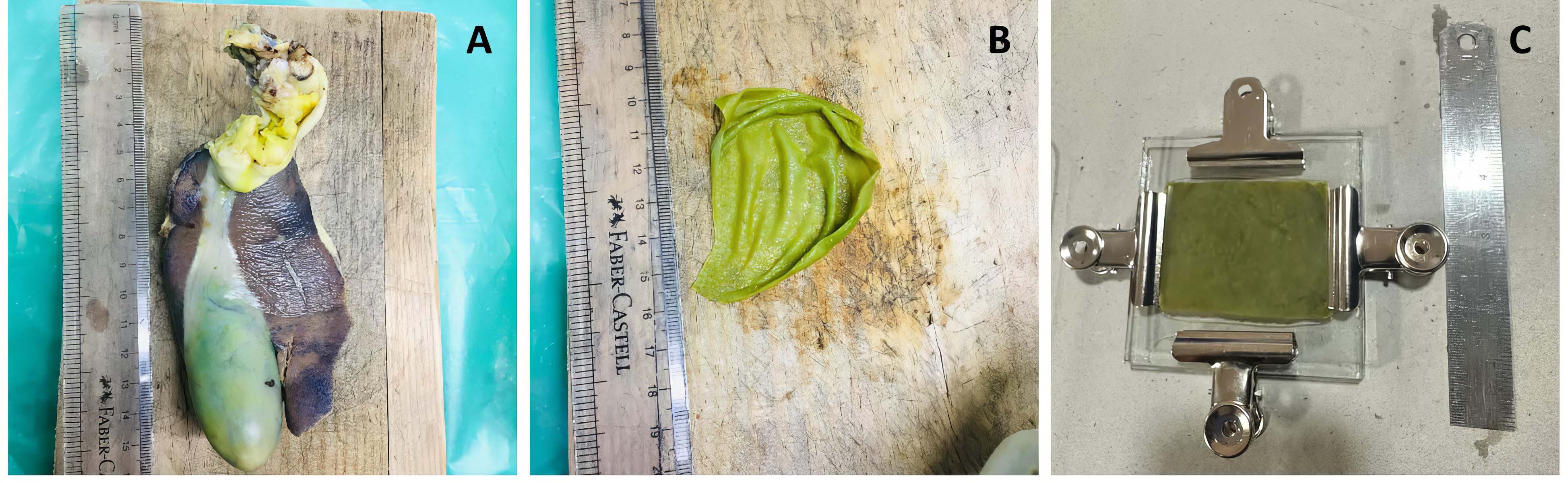

Supplement: Supplementary file 1 — Appendix Figure 1 [file 41405_2025_370_MOESM1_ESM.jpg]

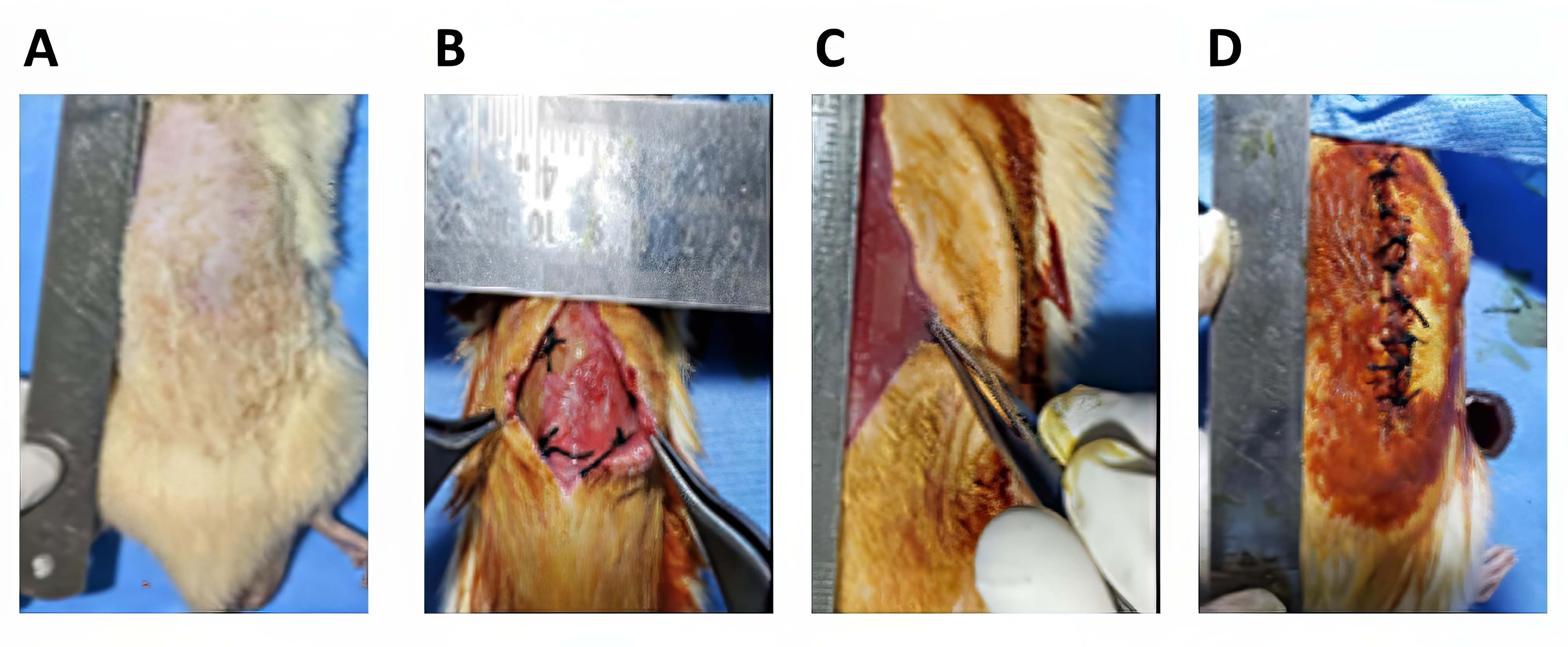

Supplement: Supplementary file 2 — Appendix Figure 2 [file 41405_2025_370_MOESM2_ESM.jpg]
