# Supplementary material for: In vitro and in vivo biocompatibility of a porcine cholecystic extracellular matrix (CECM) membrane for tissue regeneration
Source: BDJ Open. 2025 Oct 9;11:81. doi: 10.1038/s41405-025-00370-4 (PMC12511280; doi:10.1038/s41405-025-00370-4)
Supplement: Supplementary file 3 — Appendix Figures legends [file 41405_2025_370_MOESM3_ESM.docx]

**Appendix Figure 1:** Fabrication process of porcine CECM-based GTR membrane. (**A**) Fresh gallbladder preparation and bile removal; (**B**) Decellularization and layer separation; (**C**) Membrane processing, lyophilization, and sterilization. Scale bars: 1 cm.

**Appendix Figure 2:** Schematic diagram of the subcutaneous implantation procedure showing: (**A**) anatomical location of the dorsal incision; (**B**) creation of the subcutaneous pocket; (**C**) placement and positioning of the scaffold (10 mm × 10 mm × 0.5 mm); and (**D**) incision closure technique. Scale representations and anatomical landmarks are included to ensure surgical reproducibility.
